# Do Sprinters and Jumpers Differ in the Acute Countermovement-Jump Response to Isometric Conditioning Protocols? An Exploratory Study

**DOI:** 10.3390/muscles5030067

**Published:** 2026-09-18

**Authors:** Atila Salaj, Hrvoje Ajman, Frane Žuvela, Petar Šušnjara

**Affiliations:** 1 Faculty of Kinesiology Osijek, Josip Juraj Strossmayer University of Osijek, 31000 Osijek, Croatia; atila.salaj@kifos.hr (A.S.); hrvoje.ajman@kifos.hr (H.A.); petar.susnjara@kifos.hr (P.Š.); 2 Faculty of Kinesiology, University of Split, 21000 Split, Croatia; 3 Research Group “Athletics and Kinesiological Development of Children and Youth”, Faculty of Kinesiology, University of Split, 21000 Split, Croatia

**Keywords:** conditioning activity, countermovement jump, individual response, post-activation performance enhancement, track and field

## Abstract

Sprinters and jumpers differ in their force–velocity demands, so this exploratory study compared their acute countermovement jump (CMJ) response to isometric conditioning. Methods: Twenty-two competitive track and field athletes (10 sprinters, 12 jumpers; 19.1 ± 3.3 y) were alternately allocated from a randomised list to a 3 × 5 s isometric knee-extension or isometric half-squat (*n* = 11 each). Discipline was not manipulated, and no control condition was included. CMJ height was assessed from flight time by frame-by-frame video analysis, with take-off and landing timestamps read to the nearest 0.01 s. The best of three jumps was the primary trial summary. Percentage change (ΔCMJ) was the primary outcome. Results: Sprinters increased (+2.40 ± 4.46%) whereas jumpers decreased (−2.02 ± 6.44%). Discipline and protocol were analysed jointly in one additive model. The protocol contrast was small and imprecise (−0.25 percentage points [ISO-SQ relative to IKE], 95% CI −5.50 to 5.00; *p* = 0.921). The unadjusted discipline difference was non-significant (4.42 percentage points, 95% CI −0.46 to 9.30; *p* = 0.074) and the parallel Mann–Whitney U test was borderline and implementation-dependent (*p* = 0.043–0.050). After age adjustment it attenuated to +2.51 percentage points (95% CI −2.65 to 7.66; *p* = 0.320), whereas age remained associated with ΔCMJ (−0.86% per year; *p* = 0.042). Post hoc averaged trial summaries gave larger contrasts. Conclusions: The primary comparison was inconclusive and materially attenuated by age adjustment but directionally consistent across trial summaries and protocols. The observed discipline-dependent pattern is hypothesis-generating and requires confirmation in adequately powered, controlled trials.

## 1. Introduction

Post-activation performance enhancement (PAPE) describes an acute, transient improvement in voluntary explosive performance that follows a high-intensity conditioning activity. It is conceptually and mechanistically distinct from post-activation potentiation (PAP), the short-lived increase in electrically evoked twitch force attributed largely to phosphorylation of the myosin regulatory light chains and a consequent rise in calcium sensitivity, which decays within a few minutes [1,2,3,4]. At the level of the sarcomere, phosphorylation of the regulatory light chains by myosin light chain kinase displaces the myosin heads away from the thick-filament backbone and toward actin, raising the Ca^2+^ sensitivity of the myofilaments so that a given calcium transient generates greater cross-bridge force, most clearly at the submaximal activation levels characteristic of voluntary muscle actions [5,6]. This mechanism is fibre-type dependent: fast-twitch (type II) fibres express higher myosin light chain kinase activity [6] and a greater capacity for regulatory light chain phosphorylation. On this basis, greater twitch potentiation would be expected in muscle with a higher proportion of type II fibres, although the reported magnitude of this fibre-type difference varies across studies and testing conditions, and evidence linking fibre-type distribution to potentiation should be distinguished from evidence concerning myosin light-chain kinase activity itself [7]. The term PAPE has been adopted to describe the voluntary, performance-level phenomenon, which is thought to depend on a broader set of factors, including increased muscle temperature, altered muscle–water content, and possibly altered neural drive, in addition to any residual myosin phosphorylation [1,8,9,10,11]. Direct evidence of enhanced motor-unit recruitment following a conditioning activity remains limited. Isometric conditioning activities have attracted interest as a practical alternative to isotonic exercises because they are proposed to involve a lower metabolic cost and less peripheral fatigue than equivalent dynamic efforts, although direct matched comparisons of these properties are scarce. They also require minimal equipment and are readily applied in field settings [12,13,14,15]. Maximal voluntary isometric squat variants, including bodyweight and loaded squats, pushing against an immovable bar in a back-squat position, and isometric half-squats, have been reported to increase countermovement jump (CMJ) height in trained populations, although the evidence is heterogeneous and several studies report negligible or even negative acute effects, underscoring the methodological variability of this approach [16,17,18,19,20].

A consistent feature of the PAPE literature is the large inter-individual variability of the response. The balance between potentiation and fatigue, and therefore the net performance outcome, is modulated by training status, strength level, muscle fibre-type distribution, conditioning activity intensity and volume, and the recovery interval before the explosive task [2,12,21,22,23,24,25,26].

Sprinters and jumpers, although both classified as explosive track and field athletes, present distinct neuromuscular and biomechanical profiles. Sprinting is dominated by horizontally oriented force production across repeated stretch–shortening cycles [27], whereas the take-off phase of jumping events emphasises a high vertical force impulse [28], in which lower-limb and tendon stiffness play a central role [29]. Given these differences, the balance of potentiation and fatigue produced by an isometric conditioning activity, and hence the acute CMJ response, may plausibly differ between the two groups.

Therefore, the aim of this exploratory study was to compare the acute change in CMJ height following brief isometric conditioning between competitive sprinters and jumpers. Given the exploratory design and the absence of prior evidence in this specific population, no directional hypothesis was specified.

## 2. Results

### 2.1. Participant Characteristics

Descriptive characteristics of the two disciplines are presented in Table 1. Jumpers were on average 2.45 years older than sprinters (95% CI −0.20 to 5.10; Hedges g = 0.76) and had a higher baseline CMJ (+3.63 cm, 95% CI −3.39 to 10.65; g = 0.44); neither difference reached statistical significance (age *p* = 0.068; baseline CMJ *p* = 0.294), but both are of a magnitude relevant as potential confounders of the discipline comparison (Section 3.1). Four of ten sprinters and four of twelve jumpers were under 18 years of age.

### 2.2. Measurement Reliability

Intra-rater reliability of the digitising procedure, assessed by re-analysing all 132 recorded jumps on two occasions, was excellent (ICC = 0.98 (95% CI 0.98–0.99), SEM = 1.02 cm, CV = 2.5%, MDC_95_ = 2.82 cm) with no significant systematic difference between passes (mean difference −0.18 cm, *p* = 0.151). For pre-conditioning jumps considered alone, the CV was 3.0% (SEM = 1.21 cm; MDC_95_ = 3.35 cm). Within-session trial-to-trial reliability of the three pre-conditioning CMJs (all 22 athletes) was good: ICC = 0.96 (95% CI 0.91–0.98), SEM = 1.78 cm, within-subject CV = 4.2%, MDC_95_ = 4.94 cm (≈12.2% of the grand mean), with no systematic trial-order effect (F(2.42) = 0.45, *p* = 0.64).

### 2.3. Protocol Comparison and Allocation

Athletes were distributed across the two track and field event groups and two protocols as shown in Table 2. The two protocols produced almost identical mean responses (IKE +0.51 ± 5.57%; ISO-SQ −0.53 ± 6.53%); mean difference +1.04 percentage points, 95% CI −4.36 to 6.45, with no significant difference between them (Welch t = 0.40, *p* = 0.690). Because no equivalence margin was prespecified, the confidence interval remains compatible with protocol differences in either direction, including protocol effects at least as large as the discipline contrast of interest. This interval is reported as a result in its own right: it does not establish exchangeability between protocols, and the discipline comparison was not made conditional on it. Discipline and protocol were instead analysed jointly in a single additive model (Section 2.4). The implications of the absence of stimulus quantification are considered in Section 3.1.

### 2.4. Discipline Comparison

Sprinters showed a mean increase in CMJ height of +2.40 ± 4.46% after the conditioning protocol, whereas jumpers showed a mean decrease of −2.02 ± 6.44% (Table 3). The between-group difference of 4.42 percentage points (95% CI −0.46 to 9.30) was not significant on the primary Welch *t*-test (t = 1.89, *p* = 0.074). The parallel Mann–Whitney U test was borderline and sensitive to the test implementation. The two-sided exact procedure in TIBCO Statistica gave U = 29.5 (complementary U = 90.5), *p* = 0.043, whereas the asymptotic approximation gave *p* = 0.044 without continuity correction and *p* = 0.048 with it, and an exact implementation that resolves the tied U statistic in the opposite direction gives *p* = 0.050. Across implementations the two-sided *p*-value ranged from 0.043 to 0.050. Given the numerous ties in the outcome, this result should not be read as a robust significant counterpart to the primary Welch test. The parametric standardised effect was Cohen’s *d* = 0.78 (95% CI −0.10 to 1.65), an interval compatible with effects ranging from trivial in the opposite direction to large, and the non-parametric effects were rank-biserial r = 0.51 and probability of superiority A = 0.75, the latter indicating that a randomly selected sprinter had an estimated 75% probability of a greater observed response than a randomly selected jumper, with tied pairs assigned half weight. Within-group changes on raw jump height did not reach significance (sprinters: t = 1.86, *p* = 0.096, d_z = 0.59; jumpers: t = −1.37, *p* = 0.198, d_z = −0.40). On the percentage scale, the mean within-group changes were +2.40% (95% CI −0.79 to 5.59) for sprinters and −2.02% (95% CI −6.11 to 2.08) for jumpers.

The direction of the discipline difference was preserved within both protocols (IKE: sprinters +3.44 ± 5.08% (*n* = 6) vs. jumpers −3.00 ± 4.12% (*n* = 5); ISO-SQ: sprinters +0.84 ± 3.38% (*n* = 4) vs. jumpers −1.32 ± 7.96% (*n* = 7)). The complete two-way ANOVA (Type III sums of squares) yielded discipline F(1.18) = 2.86, *p* = 0.108, partial η^2^ = 0.137; protocol F(1.18) = 0.03, *p* = 0.858, partial η^2^ = 0.002; and discipline × protocol interaction F(1.18) = 0.71, *p* = 0.410, partial η^2^ = 0.038. With cell sizes of 4–7, these terms are estimated imprecisely and cannot rule out protocol-dependent patterns. The interaction is reported as exploratory only.

The cell standard deviations were 5.08, 3.38, 4.12 and 7.96 percentage points, a 2.4-fold ratio, so the homogeneity-of-variance assumption underlying the two-way ANOVA is questionable, and formal tests of it are underpowered at these cell sizes. In the additive model omitting the interaction (ΔCMJ% ~ discipline + protocol), the discipline contrast was +4.37 percentage points (95% CI −0.90 to 9.64; *p* = 0.099) and the protocol contrast (ISO-SQ relative to IKE) was −0.25 percentage points (95% CI −5.50 to 5.00; *p* = 0.921). On the absolute scale, the between-discipline difference in change was 2.04 cm (95% CI 0.13 to 3.96; Welch t = 2.23, df = 19.6, *p* = 0.038), compared with *p* = 0.074 on the declared primary percentage scale. The corresponding two-way ANOVA discipline term was *p* = 0.068 on the centimetre scale versus *p* = 0.108 on the percentage scale. The percentage scale remains the primary outcome, and this contrast is reported to make the scale dependence explicit rather than to displace the primary analysis. Age was associated with ΔCMJ (Pearson r = −0.529, *p* = 0.011), an association estimated more precisely than the discipline contrast itself, and the gradient was monotonic across age bands (+2.65% at 16–17 years, *n* = 8; −0.45% at 18–19 years, *n* = 8; −2.97% at 20–29 years, *n* = 6). In the age-adjusted additive model (ΔCMJ% ~ discipline + protocol + age), the discipline contrast attenuated from +4.37 to +2.51 percentage points (95% CI −2.65 to 7.66; *p* = 0.320), while age remained associated with the outcome (β = −0.86% per year, 95% CI −1.68 to −0.03; *p* = 0.042) and the protocol coefficient remained small and imprecise (+1.05 percentage points [ISO-SQ relative to IKE]; *p* = 0.664). Discipline and age were only partially collinear (point-biserial r = −0.380), so the two are separable in principle, but at *n* = 22 all coefficients are imprecisely estimated, the model is unstable, and residual confounding by unmeasured variables cannot be excluded. Under the post hoc mean-of-three summary the adjusted discipline contrast remained larger (+4.98 percentage points, 95% CI 1.20 to 8.77; *p* = 0.013), which illustrates that the estimate depends materially on the chosen trial summary and reinforces the separation of the primary best-of-three analysis from post hoc robustness checks. Sex showed little univariable association with ΔCMJ, and baseline CMJ height was only weakly related to it. Given the sample size, these null associations should not be taken as evidence that either variable cannot confound the discipline comparison, or that regression-to-the-mean effects are absent.

### 2.5. Sensitivity Analyses of the Trial Summary

Because the maximum of three trials is an extreme-value statistic, the discipline comparison was repeated using the mean of the three trials and the mean of the two best trials (Table 4). These analyses were added after the data had been inspected and are therefore post hoc; they are robustness checks on the choice of trial summary, not independent confirmatory evidence for the primary comparison. The discipline contrast was directionally consistent and larger under both alternative summaries (mean of three: sprinters +3.19 ± 3.57% vs. jumpers −3.47 ± 4.77%, mean difference 6.67 percentage points, 95% CI 2.95 to 10.38, Welch *p* = 0.001, d = 1.56; mean of two best: +3.89 ± 4.16% vs. −2.79 ± 5.96%, mean difference 6.68 percentage points, 95% CI 2.15 to 11.20, Welch *p* = 0.006, d = 1.28). The group SDs were smaller under the averaged summaries, consistent with reduced trial-selection noise. The larger contrast under averaged summaries may itself partly reflect the interaction between extreme-value selection and within-session variability. Mean within-subject SD across the three trials increased from pre to post more in jumpers (1.32 to 1.95 cm) than in sprinters (1.89 to 2.18 cm), so a best-of-three summary can inflate post-conditioning values differently across groups, whereas averaging reduces this selection effect. These results therefore support the robustness of the direction to the choice of trial summary, and should not be read as independent evidence of a genuine discipline effect.

## 3. Discussion

The aim of this exploratory study was to compare the acute change in CMJ performance following brief isometric PAPE protocols between competitive sprinters and jumpers. The principal observation was a discipline-dependent pattern: sprinters tended to improve (+2.4%) while jumpers tended to decline (−2.0%), with a moderate-to-large standardised effect, a borderline non-parametric test, and directional consistency across two different isometric protocols and across alternative trial summaries. Taken together, these observations suggest that the acute response to isometric conditioning may not be uniform across explosive track and field athletes, while the design limits how far this pattern can be attributed to the conditioning activity itself.

The two conditioning protocols were directed at different portions of the lower-limb extensor musculature. The IKE protocol loaded the knee extensors (rectus femoris and the vasti) in relative isolation, at full knee extension and therefore at a shortened muscle length and a joint angle mechanically unfavourable for force production. The ISO-SQ protocol, performed at a 90° knee angle against an immovable barbell, engaged the extensor chain more completely. The knee extensors together with the gluteal and hamstring muscles acting at the hip, the triceps surae, and the trunk musculature stabilising against the bar. The countermovement jump itself depends on coordinated extension at the hip, knee and ankle, so ISO-SQ shares considerably more of the jumping musculature than IKE does, yet the larger discipline contrast was observed within IKE rather than ISO-SQ (Section 2.4), a pattern the present design cannot explain. Because no electromyographic, force or torque data were recorded, the muscle groups named here are those the protocols were intended to load and not muscles in which potentiation or fatigue has been demonstrated; therefore, the mean changes of +2.40% in sprinters and −2.02% in jumpers are whole-task outcomes and cannot be localised to any particular muscle group.

The primary comparison did not show precise results. The 95% CI for the discipline difference (−0.46 to 9.30 percentage points) is compatible with no difference as well as with a range of potentially meaningful differences favouring sprinters, and the study cannot discriminate reliably between these possibilities. The divergence between the Welch and Mann–Whitney results warrants caution rather than stronger inference. The two tests address different hypotheses and respond differently to distributional shape and unequal dispersion, which plausibly differ between the groups (SD 4.46 vs. 6.44; jumper Shapiro–Wilk W = 0.87, *p* = 0.06), so the borderline non-parametric result, which straddles the conventional threshold depending on the implementation used, cannot be interpreted unambiguously as evidence of a mean difference. We do not attribute the inconclusive primary result to insufficient power, as no a priori sample-size justification was performed and such post hoc reasoning is circular. The post hoc sensitivity analysis in which the discipline contrast strengthened under trial summaries less sensitive to extreme-value noise, are best read as robustness checks on the choice of trial summary rather than as independent corroboration: they were added after the data had been inspected, they share all the design limitations discussed below, and the increase in contrast under averaged summaries may itself partly reflect the differential effect of best-of-three selection given the greater pre-to-post increase in within-subject variability among jumpers (Section 2.5). The primary estimate is also sensitive to individual observations at this sample size: in a leave-one-out check the Welch *p*-value ranged from approximately 0.009 to 0.130, and omission of a single participant raised it from 0.074 to about 0.130, whereas the mean-of-three analysis was more stable. Most importantly, the discipline estimate was materially attenuated after adjustment for age (Section 2.4), so age-related confounding remains a live alternative account of the observed pattern.

The two isometric protocols produced similar mean responses, with a protocol contrast that was small and imprecisely estimated. This is consistent with, but does not establish, comparable potentiating stimuli. No force, torque or perceived-effort data were collected, so neither the maximality of the contractions nor the equivalence of the two stimulus doses can be verified, and the confidence interval for the protocol difference (−4.36 to +6.45 percentage points) remains compatible with protocol effects at least as large as the discipline contrast. Accordingly, the absence of a detected protocol main effect is not used here to validate pooling or to infer stimulus equivalence, and both factors are retained jointly in the additive model. The IKE condition in particular was performed at full knee extension with the shank unconstrained and no external resistance beyond limb weight, so the actual knee-extension moment and contraction intensity cannot be verified and no claim is made that IKE delivered a quantified maximal conditioning stimulus. Descriptively, the larger discipline contrast occurred within IKE (sprinters +3.44%, jumpers −3.00%) rather than ISO-SQ (+0.84% and −1.32%). With cell sizes of 4–7 and a non-significant interaction, this does not establish a protocol-specific mechanism, but it does reinforce that the stimulus doses are not known to be equivalent. The two-way ANOVA gave no indication that the discipline pattern was driven by protocol allocation (interaction *p* = 0.41), although this term is exploratory.

A physiologically motivated, but untested, hypothesis for the observed pattern is as follows. The net outcome of any conditioning activity reflects the balance between potentiation and fatigue [2,22,23,30], and this balance may be shaped by fibre-type distribution, strength level, and the stiffness of the muscle–tendon unit. At the muscle level, the potentiating arm of this balance is closely tied to myosin regulatory light chain phosphorylation, which is more pronounced in fast-twitch than in slow-twitch fibres [5,7], so muscle that expresses or recruits a larger fraction of type II fibres could, in principle, be better positioned to convert a brief maximal isometric stimulus into net potentiation rather than fatigue. Under this hypothesis, sprinters may have extracted a net benefit from the brief stimulus, whereas jumpers may have been shifted toward the fatigue side of the balance. We emphasise that none of the implicated variables (fibre-type distribution, phosphorylation, muscle–tendon stiffness, or maximal strength) was measured in this study, so this account is offered strictly as a hypothesis for future testing rather than as an explanation of the present data. Two features of the cited literature further constrain it: potentiation can occur without acute changes in tendon stiffness [31], which weakens interpretations based on acute stiffness modification, and the association between tendinous stiffness and contractile performance [29] does not predict a differential response between sprinters and jumpers. Alternative explanations that the present data cannot exclude include the older age and different sex distribution of the jumper group (Section 3.1) and the possibility that jumpers potentiate on a different time course, which a single post-conditioning time point 5 min after the stimulus cannot detect. The decline observed in several jumpers is consistent with reports of negligible or transiently negative jump responses to isometric conditioning at particular recovery times [17,18]. This alternative account can be made more explicit. Reference [17] reports a decline in jump height at the third and fifth minutes following a voluntary-effort isometric protocol, with a later peak, whereas reference [20] reports benefits across a different recovery window following isometric half-squats. The single post-conditioning measurement at 5 min in the present study therefore falls at a time point at which different isometric protocols may be on different trajectories, so a discipline difference in potentiation kinetics rather than in potentiation magnitude would be sufficient to produce the observed pattern. This remains a plausible account rather than one demonstrated in the present sample, since only one post-conditioning time point was sampled. A recent systematic review and meta-analysis of isometric conditioning activities provides further context [32]. Pooling 25 studies and 782 participants, that review reported a small positive effect of isometric conditioning on vertical jump height relative to control conditions and a trivial-to-small within-group pre–post improvement, with subgroup and meta-regression analyses indicating more favourable responses at recovery intervals of approximately 4–8 min. The 5 min interval used here therefore falls within, rather than outside, the window generally associated with the largest pooled effects, so simple mistiming is an insufficient explanation on its own; protocol-specific differences in the trajectory of the response remain the more plausible reading. Two further observations from that review bear on the present protocols. Larger pooled effects were associated with total contraction durations of approximately 8–12 s and moderate session volumes, whereas both protocols here delivered 15 s of instructed isometrics contraction, although contraction intensity was not quantified and maximality could not be verified, particularly in the IKE condition. In addition, the pooled effect reached significance in participants with at least two years of strength-training experience but not in those with less, a pattern that runs counter to the age gradient observed here, in which the younger athletes showed the more favourable responses. Both observations reinforce the exploratory status of the present findings.

From an applied perspective, these exploratory observations caution against assuming that a brief isometric conditioning activity produces a uniform acute response across explosive athletes. The present data are insufficient to support discipline-specific prescription. Any practical use of such protocols should be accompanied by individual monitoring, and confirmatory evidence from controlled designs is required before recommendations can be made.

### 3.1. Limitations

This study has limitations that define the appropriate reading of its results. First, no no-conditioning control condition and no repeated test–retest arm were included, so the observed pre–post changes cannot be separated from effects of the repeated jumps themselves, the extended warm-up, familiarisation, time, or measurement variation; the three pre-conditioning CMJs may themselves have influenced subsequent performance in a direction and magnitude the design cannot determine. The findings are accordingly framed as an exploratory comparison of observed pre–post changes rather than as evidence that the conditioning protocols caused PAPE. Second, group-level contrasts reduce the influence of random measurement error, but aggregation does not remove systematic quantisation, frame-selection bias or other non-random measurement effects, and a criterion instrument (force plate) with ≥240 Hz recording would materially improve precision. Related to this, flight time was resolved to 0.01 s (Section 4.3), so the underlying observations are coarsely discrete: a single 10 ms increment corresponds to approximately 1.44 cm, or about 3.4% of a typical 42 cm jump. The mean changes reported for both groups (+2.40% and −2.02%) are each smaller than one such increment, and the observed ΔCMJ values contain no value between zero and approximately ±3.05%. The group means therefore summarise mixtures of relatively coarse individual step changes. The reported MDC_95_ values are individual-level indices and are not significance thresholds for a between-group mean difference; together with the quantisation, however, they indicate that many individual pre–post changes cannot be confidently distinguished from measurement and within-session variability. Inference is accordingly kept at the group and exploratory level, and no individual-response interpretation is offered. Third, the flight-time formula assumes identical body configuration at take-off and landing. Landing with greater knee and ankle flexion inflates flight time, and this bias could differ systematically between pre and post if the conditioning activity induced fatigue, offering a further alternative explanation, particularly for the apparent decline in jumpers. Fourth, all videos were digitised by a single assessor who was not blinded to discipline or condition. Intra-rater reliability was excellent, but inter-rater reliability and blinding-related bias were not assessed. Fifth, performance was sampled at a single post-conditioning time point, so discipline differences in potentiation kinetics cannot be excluded. Sixth, discipline is observational and confounded: jumpers were older (by 2.45 y on average), the sex distribution differed (5/5 vs. 8/4 M/F), training history was not recorded, and no measure of maximal strength was obtained. An exploratory age-adjusted model was therefore fitted (Section 2.4), in which the discipline contrast attenuated from +4.37 to +2.51 percentage points (*p* = 0.320) while age remained associated with the outcome. Age-related confounding is therefore not merely possible but quantitatively relevant to the central comparison, and the adjusted estimate is itself imprecise, potentially unstable at this sample size, and subject to residual confounding by variables that were not measured. Seventh, the conditioning stimulus was not quantified in either protocol—the IKE in particular was performed without external resistance and at a joint angle mechanically unfavourable for the knee extensors—so neither maximality nor stimulus equivalence can be verified, and the impulse-equating precaution of prior work [20] was not reproduced. Eighth, the warm-up run was prescribed at a fixed absolute pace, imposing different relative intensities on athletes of heterogeneous standards, which is itself a potential modulator of the subsequent response.

Ninth, the 5 s inter-repetition recovery used in both conditioning protocols was adopted from the athletes’ habitual training practice rather than from published conditioning protocols, which have generally used longer inter-repetition intervals. Shorter recovery between repetitions may shift the balance between potentiation and fatigue toward fatigue, and this parameter has not been evaluated systematically in the isometric conditioning literature. Its effect on the present results is therefore unknown. Tenth, the session included four 5 min passive rest periods, so post-conditioning testing occurred well after the running warm-up. Because muscle temperature is discussed as a possible contributor to PAPE, progressive cooling across the session is a plausible uncontrolled time-related factor. Relatedly, the preparatory exercises included three countermovement jumps before the three baseline CMJs, so baseline performance was itself preceded by jumping activity. Finally, the study was not prospectively registered, no a priori sample-size calculation was performed, and day-to-day variability of the outcome was not quantified.

### 3.2. Future Directions

Future studies should employ adequately powered, preferably crossover, designs in which each athlete completes both isometric protocols and a no-conditioning control condition, quantify the conditioning stimulus (force or torque, ideally with matched impulse), use criterion measures of jump height at ≥240 Hz or force-platform derivation, and sample multiple post-conditioning time points to capture individual potentiation kinetics. Incorporating measures of maximal strength, muscle–tendon stiffness and fibre-type proxies would allow the hypothesis proposed here to be tested directly. Whether chronic, training-induced differences in muscle phenotype, including fibre-type composition and the mitochondrial remodelling effected over months and years by biogenesis and by autophagic and mitophagic turnover, modulate the acute balance between potentiation and fatigue is at present unexplored, and would require designs pairing acute conditioning protocols with molecular and fibre-type phenotyping rather than the acute, performance-level measurements used here. Replicated intervention and control observations within the same individuals would be required to identify true individual responses [33,34]. Future confirmatory studies would also benefit from preregistration of hypotheses and analysis plans, together with open sharing of the dataset.

## 4. Materials and Methods

### 4.1. Experimental Design

A between-subjects design with a within-subject pre–post component was used. Each participant completed a single isometric conditioning protocol, and CMJ height was measured immediately before and after the protocol at a single post-conditioning time point. The two factors examined were track and field discipline (sprinters vs. jumpers) and protocol (IKE vs. ISO-SQ). These factors differ in status: protocol was randomly allocated, so the design is experimental with respect to protocol, whereas discipline is a pre-existing athlete characteristic that was neither manipulated nor randomised, so the design is observational with respect to discipline, and any discipline-related difference is associational rather than causal. No no-conditioning control group and no repeated test–retest arm were included. The primary outcome was the percentage change in CMJ height (ΔCMJ). The study was not prospectively registered, and no a priori sample-size calculation was performed; the sample comprised all eligible athletes available to the investigators.

### 4.2. Participants

Twenty-two healthy track and field athletes (13 men and 9 women; aged 16–29 years) volunteered to participate. All had at least two years of athletics training, had completed one to three strength-training sessions per week in the preceding year, were familiar with bilateral jumping, and had finished in the top five at the Croatian Championships or Croatian Cup in the previous season; 17 had represented the Croatian national team in at least one age category. Athletes were classified as sprinters or jumpers on the basis of the discipline they most often competed in during the last competition season. The primary event categories represented were pole vault (*n* = 6), high jump (*n* = 3), long jump (*n* = 2), triple jump (*n* = 1), 100 m/200 m (*n* = 4), 400 m (*n* = 2), 100 m hurdles (*n* = 2), and 400 m hurdles (*n* = 2). No athlete competed in both categories. The “Sprint” group included all running events up to 400 m, including 400 m hurdles. “Jumps” group included pole vault, high jump, long jump and triple jump. Athlete-level international results and event-specific performance standards were not systematically recorded. All participants were injury-free and in good general health at the time of testing. Participants refrained from strenuous activity, creatine supplementation, and alcohol for 48 h and from caffeine for at least 8 h before testing. All data were collected on 20 January 2024. All 22 allocated participants completed their assigned protocol and were included in the analysis. There were no withdrawals or adverse events.

### 4.3. Procedures

Before testing, all participants completed a standardised warm-up consisting of 5 min of running at 6:30 min/km followed by a set of 15 general preparatory exercises, listed in Table 5.

For conditioning protocols, two isometric protocols were used. The IKE protocol comprised an equipment-free isometric knee-extension held for 3 repetitions of 5 s with 5 s rest between the repetitions. The 3 × 5 s volume and 5 s inter-repetition recovery were adopted from the participants’ habitual training practice, in which maximal isometric contractions are performed as sets of 3–5 repetitions of 5 s separated by approximately 5 s of rest. This parameter set was chosen so that the conditioning activity would be familiar to the athletes and directly applicable in their normal training environment. A comparable 3 × 5 s maximal isometric volume, though with a longer inter-repetition interval, has been used previously [35]. Contractions in IKE protocol were performed seated on a chair with hips at approximately 90° of flexion and knees fully extended (0° of knee flexion) with the shank unconstrained. The contraction therefore acted without external resistance beyond the weight of the limb, and no force or torque was recorded. This position loaded the knee extensors (rectus femoris and vasti) in relative isolation. This arrangement departs from conventional isometric testing positions, which typically employ knee flexion of 60–90° against a fixed resistance. The protocol was developed by the authors as a deliberately minimal, equipment-free conditioning activity that could be applied field-side by coaches. The ISO-SQ protocol comprised an isometric half-squat performed against an immovable Olympic barbell, also held for 3 repetitions of 5 s with 5 s rest between the repetitions. The barbell was fixed under the safety pins of a squat rack at a height adjusted individually to each participant’s half-squat position, and the 90° knee angle was set and verified with a goniometer for every participant before the contraction. This position loaded the lower-limb extensor chain more completely, including the knee extensors together with the gluteal and hamstring muscles acting at the hip, the triceps surae, and the trunk musculature stabilising against the bar. No force output was recorded in either protocol but participants were instructed to give their maximal effort.

Each athlete was allocated to a protocol using list randomisation via the random.org website (Randomness and Integrity Services Ltd., Dublin, Ireland; https://www.random.org). The full participant list was randomised once, and athletes were then assigned alternately in the resulting order (first to IKE, second to ISO-SQ, and so on), yielding balanced protocol groups of 11. Allocation was therefore constrained to equal group sizes rather than simple randomisation, and it was not stratified by discipline, which produced a mild discipline imbalance across protocols (Table 2). Allocation was not concealed, and neither participants nor investigators were blinded. To ensure maximal motivation during testing, every athlete was verbally encouraged to give their best effort. Each athlete performed one protocol only. Athletes were instructed to wake up no later than 3 h before their designated time of testing. All 22 athletes were tested on a single date using one video-recording station and one conditioning area, with a single assessor performing all video digitising. Athletes attended in batches of five to six and began the session in a staggered order within each batch, so that sessions overlapped both within and across batches. The stagger interval was not formally recorded, and the elapsed time between the warm-up and post-conditioning testing was therefore not documented for individual athletes. The fixed inter-phase intervals of the protocol were adhered to for each participant.

**Figure 1:**
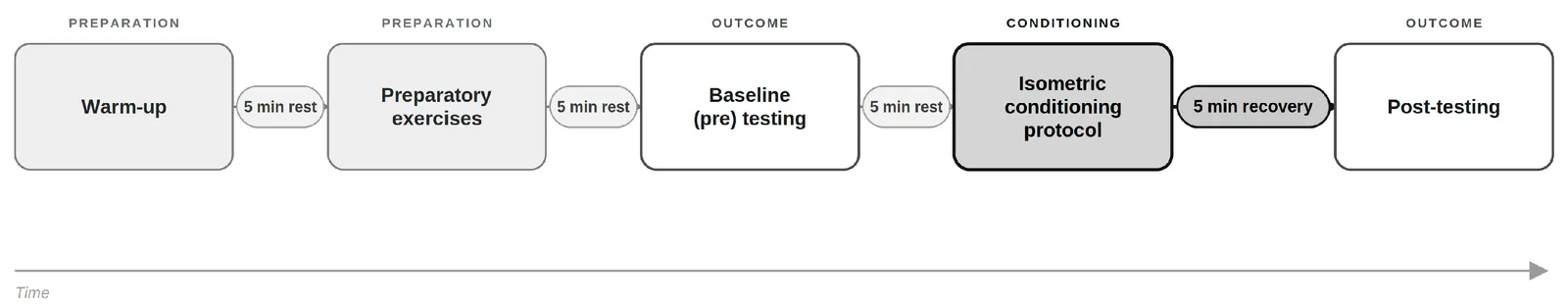
Experimental session timeline. Following a standardised warm-up and general preparatory exercises, baseline (pre-conditioning) countermovement jump performance was assessed. Participants then performed the isometric conditioning protocol, followed by a 5 min recovery interval before post-conditioning testing. All inter-phase intervals consisted of 5 min of passive rest.

Following a standardised warm-up and general preparatory exercises, baseline (pre-conditioning) countermovement jump performance was assessed. Participants then performed the isometric conditioning protocol, followed by a 5 min recovery interval before post-conditioning (final) testing. All inter-phase intervals consisted of 5 min of passive rest. Total elapsed time from the end of the running warm-up to post-conditioning testing was not formally measured but was approximately 30 min. A clear session timeline is presented in Figure 1.

Three CMJs with arms fixed at the hips and self-selected countermovement depth were performed before and after the conditioning protocol. The best of the three values was retained as the primary trial summary. Jumps within each set of three were separated by a short, unstandardised rest no longer than 5 s during which participants were preparing their next jump. Post hoc sensitivity analyses were additionally performed using the mean of the three trials and the mean of the two best trials. The CMJ was selected to minimise coordination demands and allow maximal effort. Each jump was recorded with a Xiaomi Redmi Note 9 Pro smartphone (Xiaomi Corporation, Beijing, China), configured to record at 120 frames per second, and flight time was identified in Kinovea (v0.9.5, open-source software; Joan Charmant & Contributors, Bordeaux, France; https://www.kinovea.org). All videos were digitised by a single assessor, who was not blinded to discipline or to pre/post condition. Take-off was defined as the first frame showing visible loss of foot–ground contact and landing as the first frame of visible ground contact. Flight time was obtained as the difference between the take-off and landing timestamps displayed by Kinovea, which reports time to the nearest 0.01 s. Because flight time was resolved to 0.01 s, the reported jump heights consequently lie on a 10 ms grid. The camera was configured to record at 120 frames per second; however, the original video files were not retained beyond the analysis period, and the effective decoded frame rate of the analysed files and the frame-rate setting applied in Kinovea could not be verified retrospectively. Because flight time was obtained as the difference between two independently rounded timestamps, the resulting error is not a single 10 ms quantisation of flight time: each endpoint carries a rounding error of up to ±5 ms in addition to frame quantisation. At the configured capture rate this corresponds to a flight-time standard deviation of approximately 5.3 ms, or approximately 0.76 cm at a typical jump height of 42 cm. Kinovea has demonstrated validity and reliability for spatial measurements [36]. Direct validation of the complete method used here (Kinovea, smartphone recording, timestamp readout at 0.01) is, to our knowledge, unavailable. For this reason no precision claim in this manuscript rests on nominal temporal resolution, and measurement error was instead quantified empirically (Section 2.2). Jump height was derived from flight time (t) as h = g·t^2^/8, where g = 9.81 m·s^−2^. This formula assumes an identical body configuration at take-off and landing.

### 4.4. Statistical Analysis

Analyses were performed in TIBCO Statistica v14.2.0.18 (TIBCO Software Inc., Palo Alto, CA, USA). Given the small sample, the study was treated as exploratory, and findings are interpreted primarily through effect sizes and confidence intervals. The percentage change in CMJ was calculated as ΔCMJ = (CMJ_post − CMJ_pre)/CMJ_pre × 100. The distribution of ΔCMJ within each discipline was checked with the Shapiro–Wilk test (sprinters W = 0.95, *p* = 0.66; jumpers W = 0.87, *p* = 0.06). At these sample sizes and with the outcome heavily tied because flight time was resolved to 0.01 s, the Shapiro–Wilk results carry little diagnostic weight and were not treated as confirmation of normality. The borderline result in the jumper group motivated parallel non-parametric analyses. Both parametric and non-parametric results are accordingly interpreted primarily through effect estimates and confidence intervals rather than through whether individual *p*-values cross 0.05. Cohen’s *d* is reported as a descriptive standardised mean difference, with the imprecision of its estimation at this sample size made explicit; 95% confidence intervals for d were obtained from the noncentral t distribution.

Measurement reliability was quantified at two levels. Intra-rater (digitising) reliability was assessed by re-analysing all recorded jumps on a second occasion by the same assessor, independently of first-pass data. Agreement between passes was quantified with a two-way mixed-effects, absolute-agreement, single-measures intraclass correlation coefficient (ICC) with 95% CI, together with the typical error (standard error of measurement, SEM), the coefficient of variation (CV%), and the minimal detectable change (MDC_95_ = 2.77 × SEM) [37,38]. Within-session trial-to-trial reliability was estimated from the three pre-conditioning CMJs using the same indices. Individual trial values for all participants are provided in Appendix A.

Because protocol was randomly allocated and forms part of the design, discipline and protocol were analysed jointly in a single additive linear model (ΔCMJ% ~ discipline + protocol), and the discipline estimate is reported with its 95% confidence interval. The discipline comparison was not made conditional on a preliminary test of the protocol contrast: a non-significant difference test does not establish exchangeability or equivalence between protocols, and responses were therefore not pooled on that basis.

The unadjusted discipline comparison of ΔCMJ is additionally reported using a Welch *t*-test, with the Mann–Whitney U test reported in parallel given the borderline normality of the jumper group. The Mann–Whitney test was computed in TIBCO Statistica using the two-sided exact procedure, obtained by doubling the one-sided exact probability. Asymptotic results with continuity correction, with and without adjustment of the variance for ties, are additionally reported as implemented in that software. Because ties are numerous, the fractional U statistic cannot be located exactly on the integer support of the null distribution, so exact and asymptotic procedures are not interchangeable and implementations differ in how this is resolved. The standardised effect size was expressed as Cohen’s *d* for the parametric comparison and as the rank-biserial correlation and the probability of superiority for the non-parametric comparison. Within-group pre–post changes were examined on raw jump height (cm) with paired *t*-tests and Wilcoxon signed-rank tests and reported with the standardised mean change (d_z). Percentage-scale mean changes are additionally reported with 95% CIs to keep the primary outcome scale explicit. The percentage scale is the declared primary outcome. The between-discipline contrast on the absolute (centimetre) scale is additionally reported as a descriptive sensitivity analysis to make the scale dependence of the comparison transparent. An additive model additionally including age (ΔCMJ% ~ discipline + protocol + age) was fitted to quantify the extent to which the discipline estimate is attenuated by adjustment for age. To examine whether the discipline pattern was an artefact of protocol allocation, a two-way (discipline × protocol) ANOVA with Type III sums of squares was conducted, and the complete table including the discipline main effect is reported with partial η^2^. The discipline × protocol interaction is treated as explicitly exploratory, because cell sizes are only 4–7. Because the maximum of three trials is an extreme-value statistic whose upward bias depends on within-session variability, post hoc sensitivity analyses repeated the discipline comparison using the mean of the three trials as well as the mean of the two best trials. These summaries were added after the data had been inspected and are labelled post hoc throughout. Systematic trial-order effects across the three pre-conditioning trials were examined with a one-factor repeated-measures ANOVA.

### 4.5. Ethical Approval

The study was conducted in accordance with the Declaration of Helsinki and was approved by the Ethics Committee of the Faculty of Kinesiology, University of Osijek (ethics approval number: 2158-110-01-23-32; date of approval: 13 December 2023). Written informed consent was obtained from all participants, and for participants under 18 years of age, from their legal guardians together with the participant’s assent. The approved consent covered storage, retention and analysis of all data gathered from testing, but did not explicitly address video recording or storage. To further protect participants’ privacy, the authors recorded only the participants’ lower legs, with their faces not visible in the recordings, as this was the only body region necessary for the video analysis.

## 5. Conclusions

In this exploratory uncontrolled comparison, competitive sprinters showed a more favourable observed acute CMJ response to brief isometric conditioning than jumpers. The primary between-group test was inconclusive, and the discipline estimate was materially attenuated after adjustment for age, so the comparison remains susceptible to age-related confounding. The direction of the contrast was nonetheless consistent across protocols and strengthened under post hoc trial summaries that are less sensitive to measurement noise. These observations are hypothesis-generating: they motivate adequately powered, controlled, multi-time-point trials with quantified conditioning stimuli, and they do not yet support discipline-specific practical recommendations.

## Figures and Tables

**Table 1:** Participant characteristics by discipline (mean ± SD).

Variable	Sprinters (*n* = 10)	Jumpers (*n* = 12)	Total (*n* = 22)
Sex (M/F)	5/5	8/4	13/9
Age (years)	17.8 ± 1.3	20.2 ± 4.0	19.1 ± 3.3
Age range (years)	16–20	16–29	16–29
Participants < 18 y, n	4	4	8
Body height (cm)	177.1 ± 3.5	180.3 ± 7.1	178.9 ± 5.9
Body mass (kg)	64.9 ± 4.6	67.7 ± 7.4	66.4 ± 6.3
CMJ baseline (cm)	39.99 ± 7.09	43.61 ± 8.69	41.97 ± 8.03

CMJ, countermovement jump. Between-discipline differences (jumpers − sprinters): age +2.45 y (95% CI −0.20 to 5.10; Hedges g = 0.76); baseline CMJ +3.63 cm (95% CI −3.39 to 10.65; g = 0.44).

**Table 2:** Allocation of participants to discipline × protocol cells (n).

Discipline	IKE	ISO-SQ	Total
Sprinters	6	4	10
Jumpers	5	7	12
Total	11	11	22

IKE, isometric knee-extension; ISO-SQ, isometric half-squat against an immovable barbell.

**Table 3:** Countermovement jump responses by discipline.

Group	CMJ_pre (cm)	CMJ_post (cm)	ΔCMJ (%)	ΔCMJ 95% CI (%)
Sprinters (*n* = 10)	39.99 ± 7.09	41.04 ± 7.89	+2.40 ± 4.46	−0.79 to 5.59
Jumpers (*n* = 12)	43.61 ± 8.69	42.63 ± 8.40	−2.02 ± 6.44	−6.11 to 2.08
Statistic	*p* = 0.294 ^1^	*p* = 0.654 ^1^	*p* = 0.074 ^2^/0.043 ^3^	d = 0.78; r_rb = 0.51; A = 0.75

ΔCMJ, percentage change in CMJ height; CMJ, countermovement jump. Values are mean ± SD. ^1^ Between-group comparison of raw heights by Welch’s *t*-test (*n* = 10 vs. *n* = 12; df ≈ 20). ^2^ Between-group comparison of ΔCMJ by Welch’s *t*-test, t(19.4) = 1.89. ^3^ Mann–Whitney U test, U = 29.5, n_1_ = 10, n_2_ = 12; two-sided exact *p* = 0.043 (Statistica), asymptotic *p* = 0.048 with continuity correction; an alternative exact implementation gives *p* = 0.050. d, Cohen’s *d*; r_rb, rank-biserial correlation; A, probability of superiority. Between-group mean difference = 4.42% (95% CI −0.46 to 9.30).

**Table 4:** Discipline comparison.

Trial Summary	Sprinters (%)	Jumpers (%)	Welch *p*	M–W *p*	*d*
Best of three (primary)	+2.40 ± 4.46	−2.02 ± 6.44	0.074	0.043–0.050	0.78
Mean of three	+3.19 ± 3.57	−3.47 ± 4.77	0.001	0.003	1.56
Mean of two best	+3.89 ± 4.16	−2.79 ± 5.96	0.006	0.011	1.28

Values are mean ± SD of ΔCMJ (%). Welch *p*, Welch *t*-test; M–W *p*, Mann–Whitney U test (two-sided exact); d, Cohen’s *d*, with 95% CIs from the noncentral t distribution: best of three 0.78 (−0.10 to 1.65); mean of three 1.56 (0.70 to 2.78); mean of two best 1.28 (0.43 to 2.42). Mean differences (sprinters − jumpers) with 95% CIs: best of three 4.42 percentage points (−0.46 to 9.30); mean of three 6.67 (2.95 to 10.38); mean of two best 6.68 (2.15 to 11.20). The best-of-three comparison is the primary analysis; the averaged summaries were added after data inspection and are post hoc.

**Table 5:** General preparatory exercises.

Exercise	Repetitions
Neck side bends	5
Neck flexion and extension	5
Shoulder rolls	5
Arm circles	5
Trunk rotations	5
Trunk flexion and extension	5
Trunk side bends	5
Hip circles	5
Standing quadriceps stretch	5 × 1 s
Deep split-squat	5
Lateral lunge	5
Single-leg balance (gymnastic “scale”)	5
Knee circles	5
Standing hip abduction (with support)	5
Countermovement jump	3

Values indicate the number of repetitions of each exercise; 5 × 1 s = five repetitions with a one-second hold.

## Data Availability

The data presented in this study are available on request from the corresponding author.

## Abbreviations

The following abbreviations are used in this manuscript

Abbreviation	Definition
PAPE	Post-activation performance enhancement
PAP	Post-activation potentiation
CMJ	Countermovement jump
IKE	Isometric knee-extension
ISO-SQ	Isometric half-squat
ΔCMJ	Percentage change in countermovement jump height

## Appendix A

**Table A1:** Individual trial values for all participants.

	Protocol	Sex	Group	Age	CMJ1_pre	CMJ2_pre	CMJ3_pre	CMJ1_post	CMJ2_post	CMJ3_post
1.	IKE	F	Sprint	16–17	37.09	35.76	38.46	37.09	38.46	37.09
2.	IKE	F	Jumps	16–17	41.25	39.84	42.69	39.84	41.25	37.09
3.	IKE	M	Jumps	18–19	47.14	47.14	48.67	47.14	45.63	44.15
4.	IKE	F	Sprint	16–17	33.16	30.66	30.66	33.16	30.66	34.45
5.	IKE	M	Sprint	16–17	31.89	35.76	37.09	33.16	38.46	39.84
6.	IKE	M	Jumps	20–29	44.15	47.14	44.15	48.67	44.15	45.63
7.	IKE	M	Sprint	18–19	44.15	39.84	39.84	45.63	38.46	45.63
8.	IKE	F	Jumps	20–29	27.09	25.95	24.83	24.83	24.83	24.83
9.	IKE	F	Sprint	18–19	23.74	25.95	28.25	25.95	27.09	27.09
10.	IKE	M	Jumps	16–17	42.69	39.84	39.84	38.46	41.25	39.84
11.	IKE	M	Sprint	16–17	41.25	39.84	45.63	45.63	44.15	50.23
12.	ISO-SQ	M	Jumps	20–29	55.05	55.05	51.81	50.23	47.14	47.14
13.	ISO-SQ	M	Sprint	20–29	51.81	50.23	50.23	51.81	51.81	48.67
14.	ISO-SQ	M	Jumps	20–29	53.42	51.81	53.42	50.23	55.05	50.23
15.	ISO-SQ	M	Jumps	16–17	47.14	47.14	47.14	44.15	44.15	41.25
16.	ISO-SQ	M	Jumps	18–19	45.63	45.63	45.63	41.25	45.63	47.14
17.	ISO-SQ	M	Jumps	18–19	47.14	42.69	45.63	44.15	41.25	41.25
18.	ISO-SQ	M	Sprint	18–19	44.15	47.14	44.15	42.69	47.14	48.67
19.	ISO-SQ	F	Jumps	20–29	38.46	33.16	35.76	35.76	31.89	33.16
20.	ISO-SQ	F	Sprint	18–19	34.45	37.09	37.09	35.76	38.46	37.09
21.	ISO-SQ	F	Sprint	18–19	34.45	37.09	34.45	33.16	34.45	35.76
22.	ISO-SQ	F	Jumps	16–17	25.95	28.25	28.25	25.95	31.89	30.66
